# Tree shape‐based approaches for the comparative study of cophylogeny

**DOI:** 10.1002/ece3.5185

**Published:** 2019-05-29

**Authors:** Mariano Avino, Garway T. Ng, Yiying He, Mathias S. Renaud, Bradley R. Jones, Art F. Y. Poon

**Affiliations:** ^1^ Department of Pathology and Laboratory Medicine Western University London Ontario Canada; ^2^ BC Centre for Excellence in HIV/AIDS Vancouver British Columbia Canada; ^3^ Department of Applied Mathematics Western University London Ontario Canada

**Keywords:** coevolution, cophylogeny, host switching, kernel, tree measures, tree shape

## Abstract

Cophylogeny is the congruence of phylogenetic relationships between two different groups of organisms due to their long‐term interaction. We investigated the use of tree shape distance measures to quantify the degree of cophylogeny. We implemented a reverse‐time simulation model of pathogen phylogenies within a fixed host tree, given cospeciation probability, host switching, and pathogen speciation rates. We used this model to evaluate 18 distance measures between host and pathogen trees including two kernel distances that we developed for labeled and unlabeled trees, which use branch lengths and accommodate different size trees. Finally, we used these measures to revisit published cophylogenetic studies, where authors described the observed associations as representing a high or low degree of cophylogeny. Our simulations demonstrated that some measures are more informative than others with respect to specific coevolution parameters especially when these did not assume extreme values. For real datasets, trees’ associations projection revealed clustering of high concordance studies suggesting that investigators are describing it in a consistent way. Our results support the hypothesis that measures can be useful for quantifying cophylogeny. This motivates their usage in the field of coevolution and supports the development of simulation‐based methods, i.e., approximate Bayesian computation, to estimate the underlying coevolutionary parameters.

## 1 INTRODUCTION

Coevolution occurs when two or more species exert a reciprocal influence on one another's evolutionary trajectories (Vermeij, 1994). These effects may be mediated by beneficial (mutualistic) or deleterious associations (e.g., parasitism, predation). For simplicity, we will only refer to “host” and “pathogen” species, although we recognize that many other roles in coevolutionary interactions exist in nature. A cophylogenetic study is a comparative analysis of the evolutionary relationships within sets of host and pathogen species, and the extent that these relationships are correlated back in time. Host–pathogen associations are frequently visualized by a “tanglegram,” in which the associations are mapped to the two phylogenies by drawing association edges between the respective host and pathogen taxa (Page, 1993). If the topologies of the two phylogenies are fully concordant, then there exists an arrangement of their branches (by rotation around ancestral nodes) such that the association edges do not intersect – the trees are completely “untangled.” This situation implies that the interactions between the host and pathogen species are so strong that the diversification of the pathogen species is entirely constrained by that of their hosts. Discordant trees can also yield an untangled graph. Thus, the number of intersecting association edges is a more useful measure for optimizing visual layouts than for inferring biological processes.

Any single tanglegram may be explained by a large number of different combinations of events in the past, including pathogen and/or pathogen‐mediated host extinction, host (sometimes biased host) switching, incomplete lineage sorting (Pamilo & Nei, 1988), pathogen speciation/duplication, and unobserved species; see Charleston and Perkins (2006) for a detailed discussion of these event types. Increasing numbers of events in the coevolutionary history of the host and pathogen species will tend to result in a lower degree of topological concordance between their phylogenies. Estimating the optimal reconstruction of such events to explain the present‐day associations between the tip taxa of the host and pathogen phylogenies is known as reconciliation inference (Doyon, Ranwez, Daubin, & Berry, 2011; Doyon et al., 2010). A well‐characterized approach to reconciliation inference is to assign a cost to each type of event and to identify the most parsimonious (minimum cost) distribution of events. However, the resulting solution is sensitive to the investigator's choice of costs, and becomes exceedingly difficult for larger trees. Indeed, this approach becomes a computationally intractable (NP‐hard) problem if time‐consistent reconciliation is required (Ovadia, Fielder, Conow, & Libeskind‐Hadas, 2011), so that lineage transfer events do not contradict the timings of internal nodes between the trees. This problem has been addressed by Libeskind‐Hadas and Charleston (2009), who provide algorithms for computing the set of Pareto‐optimal event counts and thereby estimate the best set of cost parameters for a particular reconciliation, and recently also by Ma, Smirnov, and Libeskind‐Hadas (2017), who adopted a combination of algorithms to efficiently find temporally feasible reconciliations. Probabilistic approaches to reconciliation, such as amalgamated likelihood estimation (Szöllősi, Rosikiewicz, Boussau, Tannier, & Daubin, 2013), can jointly estimate the costs of different cospeciation events in exchange for increased computational complexity and sensitivity to accurate scaling of branch lengths in time (Scornavacca, Jacox, & Szöllősi, 2014). Further, Bayesian reconciliation methods enable the investigator to relax the assumption that the host and parasite phylogenies are known without error (Huelsenbeck, Rannala, & Larget, 2000), and instead sample phylogenies from an appropriate prior distribution such as the birth–death model (Arvestad, Berglund, Lagergren, & Sennblad, 2003; Sjöstrand et al., 2014). Sampling two phylogenies can result in an enormous model space, however, such that the computational time required for convergence to the posterior distribution may become excessive for substantial numbers of taxa. Finally, it is not uncommon to simply visualize the tanglegram and make a qualitative, subjective assessment about the extent of cospeciation. By focusing on the association edges, this manual approach may overlook differences in the internal topologies or timescales between the two trees.

We propose to introduce distance measures of tree shapes to the field of cophylogeny, which might occupy a middle‐ground between these extremes. Specifically, our objective is to assess whether such simple quantitative methods may be useful for estimating coevolutionary parameters from differences in tree shapes. There is an abundance of distance measures for comparing trees with respect to their topology and/or branch lengths. For example, numerous investigators have proposed various summary statistics that each extract certain characteristics of tree shapes, such as asymmetry (e.g., Colless’ index) and thereby reduce the tree to a single number; for a comprehensive review, see Mooers (1997). Summary statistics provide a convenient framework for comparing trees, which are otherwise statistically complex objects. However, many of these statistics are difficult to normalize to differences in tree size (Stam, 2002), and can be strongly influenced by sampling for rapidly evolving taxa (Dearlove & Frost, 2015). In addition, the inherent dimensionality reduction of these summary statistics is often accompanied by a critical loss of information about the underlying biological processes, which can limit the utility of any one statistic. For this reason, recent studies have begun to use feature selection methods to find optimal combinations of summary statistics (e.g., Saulnier, Gascuel, & Alizon, 2017).

Whereas a summary statistic maps a tree to a number, a distance measure (sometimes referred to as a “metric”) maps two trees to a number that quantifies their level of discordance. One of the earliest distances for trees was the cophenetic correlation (Sokal & Rohlf, 1962), in which the depth of the lowest common ancestor between each pair of tips in the tree is represented by a distance matrix. The ordinary product–moment correlation for two trees is then calculated from the element‐wise comparison of their respective matrices. This method was rendered as a distance measure by Cardona, Mir, Rosselló, Rotger, and Sánchez (2013) and slightly modified by Kendall and Colijn (2016), referred here as KC (or KCw when considering branch lengths). A distance described by Williams and Clifford (1971), denoted here as “Node” (Kuhner & Yamato, 2014), restricts this correlation to the internal nodes of the trees and measures path lengths by numbers of nodes. Similarly, “pathdist” (Steel & Penny, 1993) is a path distance measure that substitutes the L2‐norm (Euclidean distance) for the L1‐norm (total absolute difference) employed by Node. More recently, Kuhner and Yamato (2014) proposed a topology‐free distance “Int” that sums the differences in inter‐node branch lengths, proceeding from the most recent tip to the root.

Other distance measures place greater emphasis on tree topologies. For instance, the Maximum Agreement Subtree (MAST; Gordon, 1980) distance is based on the largest labeled subtree that is common to both trees. The Robinson–Foulds distance (RF; Robinson & Foulds, 1981), by far the most cited tree distance in the literature (Table 1), provides symmetric distance between two phylogenies as a sum of monophyletic groups present in one tree but not in the other, given that they relate the same set of taxa. The RF distance has been also extended to consider branch lengths, either by incorporating the L1‐norm (RFL, Robinson & Foulds, 1979) or L2‐norm (KF; Kuhner & Felsenstein, 1994), and further adapted to accommodate unrooted trees (nPH85; Penny, Foulds, & Hendy, 1982; Geoghegan, Duchêne, & Holmes, 2017). Moreover, Nye, Lio, and Gilks (2005) proposed a method similar to the RF distance that takes the optimal one‐to‐one mapping of branches between the trees as a distance “Align”. Instead of shared subtrees of any size, Critchlow, Pearl, and Qian (1996) described a distance (Trip) based on triples of related taxa, which was later extended by Kuhner and Yamato (2014) to utilize branch lengths (TripL). The Billera–Holmes–Vogtmann (BHV, Billera, Holmes, & Vogtmann, 2001) distance measure captures both topology and branch lengths by mapping tree shapes into a geometric space, which can be traversed by varying branch lengths and resolving the polytomies that result from zero branch lengths. In addition, Hein, Schierup, and Wiuf (2004) proposed a distance “Sim” based on the probability that a random point in one tree is on a branch leading to the same set of tip labels in a second tree.

**Table 1:** Summary of tree distance measures examined in this study. In addition to the kernel measures kU and kL, we evaluated two additional measures where branch lengths were normalized by the mean (kUn and kLn). “Diff. size” indicates which distances do not require the trees to have the same numbers of tips. “Diff. labels” indicates which distances do not require the trees to relate the same taxa, i.e., to have the same labels. “Use lengths” indicates which distances utilize the differences in branch lengths when comparing trees. We enumerated citations in the literature by querying Google Scholar (last access date, June 31, 2017) for papers associated with the respective distance measures and software, and then filtered the results for coevolutionary studies (Coevol.). Measures pathdistw and KCw referred to pathdist and KC, respectively, with lengths enabled

Distance	References	Diff. size	Diff. labels	Use lengths	Citations	
					Total	Coevol.
RF	Robinson and Foulds (1981)				1,561	3
nPH85	(Geoghegan et al., 2017; Penny et al., 1982)			Y	227	1
Trip	Critchlow et al. (1996)	Y			103	0
MAST	Gordon (1980)		Y		69	0
Align	Nye et al. (2005)				123	1
Node	Williams and Clifford (1971)		Y		82	0
KF	Kuhner and Felsenstein (1994)			Y	725	0
Sim	Hein et al. (2004)	Y		Y	540	0
TripL	Kuhner and Yamato (2014)			Y	13	0
kU	Poon et al. (2013)	Y	Y	Y	22	0
kL	This study	Y		Y	n/a	
pathdist/pathdistw	Steel and Penny (1993)			Y	253	2
BHV	Billera et al. (2001)			Y	384	2
KC/KCw	Kendall and Colijn (2016)			Y	6	0

The majority of these distance measures can be computed efficiently, and several utilize branch lengths in addition to tree topologies (Table 1). On the other hand, most of the distances require the trees to have the same numbers of tips and the same tip labels, e.g., relating the same taxa. Many of the distance measures can also be expected to be sensitive to the placement of roots in the trees. In previous work, we proposed a new tree distance measure (Poon et al., 2013) based on a kernel function from computational linguistics (Moschitti, 2006) that essentially counts the number of isomorphic fragments shared by two trees, while penalizing fragments for their discordance in branch lengths. The resulting distance measure is normalized for differences in tree sizes and can optionally ignore tip labels, such that it can be applied to trees relating different sets of taxa.

A distance measure may be difficult to interpret without some absolute scale or reference distribution. Thus, the discordance between host and pathogen trees can also be quantified by an independence test (De Vienne et al., 2013), which evaluates the probability that an equal or shorter distance is obtained by chance given a null distribution. Hence, this test essentially maps the distance measure to a more interpretable scale. The null distribution can be either generated at random from the simulation of trees given a parametric model, or by the nonparametric permutation of the host and pathogen trees. Finally, we note that this is not a comprehensive review of distance measures on trees; we acknowledge more recent and ongoing advances in this area in the Discussion section.

Although such distance measures have been widely utilized in the comparison of trees in both evolutionary and broader contexts, there are surprisingly few references to these measures in the cophylogeny literature (Table 1). We propose that tree distance measures may provide a simple and useful complement to the visual assessment of tanglegrams or reconciliation methods, which require the investigator to either assign costs or perform intensive computation for larger data sets. In this study, our objective is to assess how much information these distance measures can extract about coevolutionary events from the discordance of host and pathogen phylogenies. This however requires evaluating these distances on sets of trees where the underlying cophylogeny process is known with absolute certainty that reconciliation methods cannot provide. Thus, we developed a reverse‐time simulation framework for generating pathogen trees along a fixed host tree, from the tips toward the root, for given rates of cospeciation, duplication, and host switching. This work provides a critical quantitative assessment on the potential utility of distance measures for cophylogenetic studies, and provides detailed guidance for choosing among those measures given prior information on the relative importance of different coevolutionary events, or to focus on specific measures that are more informative than others about coevolutionary processes. Further, we compare the distance measures to the more standard approach of reconciliation through maximum parsimony, using mutual information to contrast these methods within a consistent quantitative framework.

## 2 METHODS

### 2.1 Simulation methods

To simulate pathogen trees within host trees, we implemented a reverse‐time simulation method with a custom Python script. The required inputs of this script are: (a) a Newick string representation of the host tree, with branches scaled in units of real time; (b) the speciation rate of two pathogen lineages within the same host, Λ; (c) the migration rate for pathogen lineages between hosts (host switching), *M*, and; (d) the probability of cospeciation, *P*. The speciation of host species was a nonrandom event determined by the input tree. Stochastic events were simulated using the standard Gillespie method (Gillespie, 1977). The total rate of stochastic events was:

$$\lambda =\lambda_{P}+\lambda_{M}=\sum_{i}^{n_{h}\left(t\right)}\left(\begin{array}{c} n_{p}^{i}\left(t\right)\\ 2 \end{array}\right)\Lambda +\left\{\begin{array}{c} n_{p}\left(t\right)M\mathrm{if}\;n_{h}\left(t\right)>1\\ 0\mathrm{if}\;n_{h}\left(t\right)=1 \end{array}\right.,$$

where $n_{h}\left(t\right)$ is the number of extant host lineages at time *t*, $n_{p}\left(t\right)$ is the number of extant pathogen lineages, and $n_{p}^{i}\left(t\right)$ is the number of extant pathogen lineages within the *i*‐th host. The speciation of pathogen lineages within a host is the reverse‐time analog of a duplication event, i.e., the speciation of a pathogen lineage into two derived species within the same host lineage. We assumed that host switching was a random process that occurred at a constant and uniform rate for any single pathogen lineage. If there was only one extant host lineage at time *t*, then we assumed that the total host switching rate $\lambda_{M}$ was effectively zero.

The simulation was initialized at the most recent tips of the host tree (*t* = 0), with a single pathogen lineage assigned to each sampled host. We did not require all host species to be sampled at the same time. The heights (relative to the most recent tip at *t* = 0) of host species as determined by the input tree are denoted as $\tau_{i}\geq 0$, $i=\{1,\text{…},n_{h}^{*}-1\}$, where $n_{h}^{*}$ is the total number of tips in the host tree. Moreover, the sampling times of host species determined by the input tree are denoted $\tau_{j}^{*}$, $j=\{1,\text{…},n_{h}^{*}\}$, where $\tau_{j}^{*}=0$ for at least one value of j. The simulation was updated iteratively back in time with a random sequence of events on the timescale of the input tree. The waiting time until the next event was drawn from an exponential distribution, Δt∼exp(λ). If the waiting time exceeded the time interval to the next highest host node τ, then we updated the vector of extant host nodes and reset the simulation time. If the next highest host node was a tip, then we set $t=\tau_{j}^{*}$ and incremented $n_{p}\left(\tau_{j}\right)$ by one. Otherwise if the next highest host node was an internal node, then we set $t=\tau_{i}$. All pathogen lineages carried by the affected host lineages were transferred to the ancestral host lineage, with a cospeciation probability *P* of two randomly selected lineages from the respective derived hosts being speciated into a single ancestral lineage.

If the waiting time does not exceed the time interval to the next highest host node τ, then we determined whether the next event was a host‐switch or a within‐host speciation of pathogen lineages. If a host‐switch event occurred with probability $\lambda_{M}/\lambda$, then we selected an extant pathogen lineage at random from $n_{p}$ and reassigned this lineage to an extant host drawn at random from $n_{h}$ excluding the original host. We made the simplifying assumption that all host switching events were “complete,” in that migration to another host species was followed by speciation. A pathogen speciation event otherwise occurred with probability $\lambda_{P}/\lambda \;\left(=1-\lambda_{M}/\lambda \right)$, in which we selected a pair of lineages occupying the same host at random to speciate into a single ancestral lineage. Thus, the specific migration and speciation events were uniform across pathogen lineages and pairs of lineages, respectively. Subsequently, we incremented the simulation time *t* by the waiting time Δt and drew the next waiting time. The simulation halts when the number of extant hosts returns to one and all tips in the host tree have been sampled. If there are multiple pathogen lineages within this ancestral host, then the simulation proceeds back in time with speciation at a constant rate per pair until only one pathogen lineage remains.

We used the Python library *ete3* (Huerta‐Cepas, Serra, & Bork, 2016) to parse and construct tree objects. For each simulation we generated two different pathogen tree outputs: a tree in which branches were partitioned by nodes of degree‐size three or two to record all within‐host speciation and host‐switch events, respectively (*ete3* format 1); and a second tree in which this information was removed, leaving only internal nodes with degree‐size 3 and terminal nodes with degree‐size 1 (*ete3* format 5). Single nodes (with a single descendant) were subsequently removed with R package ape v5.0 (Paradis, Claude, & Strimmer, 2004) with function collapse.single.

### 2.2 Simulation analysis

To initialize our simulation experiments, we selected a tree relating hosts of the Hepadnaviridae (HBV) family from a recent study of host‐pathogen coevolution among DNA and RNA virus families (Geoghegan et al., 2017). The authors found that the trees corresponding to HBV and its hosts, among all the virus families analyzed, had the highest level of concordance, based on their normalized version of the Penny et al. (1982) measure (nPH85). However, the host tree Newick file published by the authors did not include any branch lengths. Consequently we obtained the time‐scaled phylogenetic tree relating Metazoa published at http://timetree.org, and pruned the tree down to the host species associated with HBV, including fish, reptiles, and amphibians (treated by the authors as one single host category), birds and mammals. Since a number of the distance measures evaluated in this study required the host and pathogen trees to share the same set of tip labels, we labeled the simulated pathogen trees by their host, and initialized simulations with only one pathogen lineage per host tip.

Using this host tree, we conducted a series of “edge case” simulation experiments in which two of the parameters were fixed to extreme values, and the third parameter was varied over a broad range (Table S1). The purpose of these edge case simulations was to provide outputs that were easy to interpret for validating the simulation model, and as a preliminary assessment of how the various distance measures responded to the model parameters. To visually inspect the simulation outputs, we plotted random samples of edge case simulations alongside the host tree with DensiTree v2.2.5 (Bouckaert, 2010). Next, we used Latin hypercube sampling to randomly generate 500 points that were evenly distributed in the parameter space. Specifically, we partitioned the range Λ=[0,1] into 500 intervals such that their midpoints were evenly spaced after a log‐transformation; we applied the same scheme to the range *M* = [0,1]. Since the parameter *P* is a probability instead of a range, we partitioned the range *P* = [0,1] into 500 intervals without any transformation. Next, we generated a random permutation of intervals independently along each axis, uniformly sampled one point within each cube defined by the intersection of three intervals, and simulated 100 pathogen trees using those parameter values for a total of 50,000 simulations. Correlations and mutual information (MI) tests on the performance and collinearity of measures were performed using the R package Entropy (Hausser & Strimmer, 2009).

### 2.3 Data collection

Here, we evaluated the distance measures on phylogenies reconstructed from actual data sets. First, we collected published data sets from the literature of host and pathogen coevolution. We queried Google Scholar (https://scholar.google.ca/) on the title and abstract fields of publications with at least one of the following search terms “concordance,” “cophylogeny” (or “co‐phylogeny”), “host,” “pathogen,” “parasite,” and “symbiont.” The number of records returned by these queries precluded an exhaustive manual curation. Consequently, we manually evaluated records returned by this query that were ranked both according to the default ordering (where articles are ranked by “relevance”, the occurrence of search terms and the number of citations in the database), and with respect to publication dates. The purpose of this dual‐ordered approach was to reduce the inherent bias of ranking articles by the number of citations, which tends to favor articles with earlier publication dates. Next, we manually reviewed, filtered, and sorted a selection of article records into two categories (Table 2): (a) studies where the reported degree of cospeciation/codivergence, based on authors’ assessment, was moderate to high, and; (b) studies where the degree was low, with phylogenies considered too difficult to reconcile due to extensive host switching, duplication, or extinction events. For each study, we obtained the sequence data using a batch query of the Genbank accession numbers. The resulting collection of 36 trees are summarized in Table 2 and are referred herein as the “General” collection.

**Table 2:** Summary of cophylogeny studies and data sets collected from the literature (the “General” collection). The keys are used to map these entries to subsequent figures. *N* denotes the number of tips in the corresponding host or parasite tree

Key	References	Host (*N*)	Pathogen (*N*)	Association
High concordance				
1–2	Mizukoshi, Johnson, and Yoshizawa (2012)	Sika deer (11)	Lice (10)	Parasitic
3–4	Duron and Noël (2016)	*Pantoea* (42)	*Ishikawaella* (42)	Mutualistic symbiosis
5–6	Kikuchi et al. (2009)	Stinkbugs (14)	Gut bacteria (14)	Symbiosis
7–8	Arai et al. (2012)	Korean crocidurine shrew (23)	Hantavirus (23)	Pathogenic
9–10	Rector et al. (2007)	Felidae (5)	Papillomavirus (5)	Parasitic
11–12	Merckx and Bidartondo (2008)	Plants (5)	Arbuscular Mycorrhizal fungus (14)	Symbiosis
19–20	Hughes, Kennedy, Johnson, Palma, and Page (2007)	Pelecaniform birds (18)	*Pectinopygus* lice (18)	Parasitic
21–22	Lanterbecq et al. (2010)	Crinoids (16)	Myzostomids (16)	Symbiosis
27–28	Hosokawa, Kikuchi, Nikoh, Shimada, and Fukatsu (2006)	Stinkbugs (7)	Gut bacteria (7)	Symbiosis
33–34	Peek, Feldman, Lutz, and Vrijenhoek (1998)	Deep sea clams (9)	Chemoautotrophic bacteria (9)	Symbiosis
35–36	Sauer, Stackebrandt, Gadau, Holldobler, and Gross (2000)	*Camponotus* (13)	Proteobacteria (13)	Symbiosis
37–38	Noda et al. (2007)	Termites (16)	Gut bacteria (16)	Symbiosis
Low concordance				
15–16	Guo et al. (2013)	Mammals (39)	Hantavirus (41)	Parasitic
17–18	Choi and Thines (2015)	Plants, compositae (61)	Downy mildews (61)	Parasitic
23–24	Santiago‐Alarcon, Rodriguez‐Ferraro, Parker, and Ricklefs (2014)	Non‐passerine birds (35)	Haemosporidian (30)	Parasitic
25–26	Hall et al. (2016)	Psyllid (20)	S‐endosymbionts (20)	Symbiosis
29–30	Lei and Olival (2014)	Bats (9)	*Bartonella* and *Leptospira* (13)	Pathogenic
31–32	Lim‐Fong, Regali, and Haygood (2008)	*Bugula* (5)	Candidatus *Endobugula* (5)	Symbiosis

Second, we obtained all of the 19 host‐virus data sets from Geoghegan et al. (2017), which we refer to as the “Viral” collection. Because the host trees in Geoghegan et al. (2017) were not available with branch lengths, we reconstructed these lengths by extracting them from time‐scaled trees published at http://www.timetree.org (Hedges, Dudley, & Kumar, 2006). We retrieved the Metazoa (*n* = 1,456 tips) and Viridiplantae (*n* = 373 tips) trees from this database at the taxonomic resolution of families. We mapped host species annotations from the virus phylogenies to these family‐level trees using the NCBI BLAST taxonomy (Sayers et al., 2009). When more than one tip in a virus phylogeny mapped to the same host family, we collapsed those tips into a single terminal branch as in Geoghegan et al. (2017). To maintain consistency with the original study, we applied midpoint rooting to the pathogen trees; however, we also evaluated outgroup rooting and placement of the root to minimize the distance to the host tree, but we found no significant effect on our results (for simplicity we restricted these tests to the kUn and kLn measures, since we would not expect scaling of branch lengths to affect sensitivity to the root).

### 2.4 Data processing

Because sequence alignments were not available from the studies in the “General” collection, we reconstructed alignments of nucleotide or amino acid sequence data using MUSCLE (version 3.8.425, Edgar, 2004) with the default settings. The resulting alignments were visually inspected and refined using AliView (Larsson, 2014). We determined the optimal substitution model for each alignment using jModelTest 2.0 (Darriba, Taboada, Doallo, & Posada, 2012) for nucleotide sequences and prottest3 (Darriba, Taboada, Doallo, & Posada, 2011) for amino acid sequences, both of which employ the Akaike information criterion for model selection. Phylogenetic trees were reconstructed by maximum likelihood using PhyML 3.0 (Guindon & Gascuel, 2003) and, for the “General” dataset, rooted on the branches determined by the respective studies. These trees were visually inspected in FigTree (Rambaut, 2012) to verify that the result was consistent with the source publications.

### 2.5 Distance measures and MP reconciliation

We used the implementation of the Robinson–Foulds (RF) distance in the R library phangorn v2.4.0 (Schliep, 2011) with following parameters: normalize = TRUE, rooted = TRUE, check.labels = FALSE. An extension of RF (KF) incorporates branch length information into the comparison of tree topologies. We used the function KF.dist in phangorn to calculate this extended measure under the default parameters, and the function path.dist with the use.weight (branch lengths) option toggled to calculate the pathdist or pathdistw measures, respectively. In addition, we used the function nPH85 in R library NELSI v0.2 (Ho, Duchêne, & Duchene, 2015) to calculate the related normalized Penny‐Hendy measure. The Billera Homes Vogtmann (BHV) measure was calculated using GeoMeTree v1.1 (Kupczok, Haeseler, & Klaere, 2008). Sokal and Rohlf's measure as Kendall and Colijn (2016) (KC) was computed with the function treeDist in the R library treespace v1.1.3, setting the optional lambda parameter to 1 to incorporate branch lengths (KCw). To calculate the measures Align, Node, MAST, Trip, and TripL in the same framework, we ported the respective implementations from the Python script published by Kuhner and Yamato (2014) into a custom R package (https://github.com/PoonLab/Kaphi). Maximum Parsimony (MP) reconciliation analyses were calculated under Duplication‐Transfer‐Loss (DTL) model, where four types of events (cospeciation, duplication, transfer, and loss) are considered and reconciliation of pathogen tree on host tree happens in forward‐time. We performed MP reconciliation analysis on the 50,000 simulated pathogen trees and HBV tree by using the software package called Cheeta (Ma et al., 2017).

A kernel function computes the inner product between two objects that have been mapped to a high‐dimensional feature space (Aizerman, Braverman, & Rozonoer, 1964). It is a highly efficient method for comparing complex objects for which there is a potentially enormous number of features in each object, because the kernel restricts its calculation to the comparable tiny subset of features that occur in at least one of the two objects. A larger inner product indicates that the objects share a greater number of features; hence, the kernel can be used as a measure of similarity. Poon et al. (2013) previously adapted a kernel function that operates on tree‐like objects in natural language processing (Collins & Duffy, 2002) to compare phylogenetic trees. The features counted by this kernel are subset trees. A subset tree is a fragment of a tree that is rooted at an ancestral node and extends down toward its descendants. It does not necessarily extend all the way to the tips of the tree – if it does, however, then it is referred to as a “subtree” (Moschitti, 2006). The tree shape kernel essentially counts the number of times that subset trees with the same topology appear in both phylogenies, and then penalizes this number by the discordance in branch lengths (Poon et al., 2013). This kernel does not utilize tip labels, so we refer to it here as the unlabeled kernel distance (kU).

Furthermore, we extended the kernel method to compare subset trees on the basis of shared tip labels. We modified the recursive function used to calculate the kernel score, by substituting an indicator function $1_{n_{1},n_{2}}$ in place of the constant 1 when the two nodes being compared are both tips (Collins & Duffy, 2002; Poon et al., 2013). The function $1_{n_{1},n_{2}}$ assumes the value 1 if *n*_1_ and *n*_2_ have the same labels, and otherwise returns 0. We refer to the resulting distance as the labeled kernel (kL). To generate kernel similarity matrices for the “General” and “Viral” data sets in this study, we first imported the Newick tree strings using the BioPython phylo module (Talevich, Invergo, Cock, & Chapman, 2012). Branch lengths were subsequently normalized by the mean branch length in each phylogeny to facilitate the comparison between host and pathogen trees with different overall rates of evolution (indicated by “n” suffix for unlabeled and labeled kernels, or kUn and kLn). The kernel scores were also normalized, using the cosine method, to adjust for differences in the overall size (number of nodes) of the respective trees (Collins & Duffy, 2002). Kernel principal components analysis and projections for the resulting matrices were generated using the kernlab package in R (Karatzoglou, Smola, Hornik, & Zeileis, 2004).

The behavior of the kernel function is controlled by several parameters. First, the branch length penalty is determined by a Gaussian radial basis function centered at zero with variance parameter σ, where a smaller σ results in a more severe penalty for subset trees with different branch lengths. Second, the kernel function includes a decay parameter λ that penalizes matching subset trees that are too large, which is useful to avoid the “large diagonal problem” (Collins & Duffy, 2002). Third, Moschitti (2006) introduced a parameter σ to control for subset tree matching, which we renamed *s* to avoid confusion with the Gaussian parameter. If *s* = 0, then matched subset trees must extend to the tips (subtrees) to be counted by the kernel. Since our trees have labels only on the tips, we fixed *s* = 0 for kL or kLn. Otherwise, the effect of labels was overwhelmed by subset tree shapes. Thus, with *s* = 1, the subset trees do not have to include all tips (kU or kUn). This parameter has especially significant importance for comparisons of labeled trees, because trees with congruent shapes and different sets of labels on their tips may be scored as highly similar when *s* = 1, and completely dissimilar when *s* = 0. Based on previous work (Poon et al., 2013), to evaluate the effect of these tuning parameters on the kernel function's sensitivity and specificity for simulated data, we initiated our analyses with the default unlabeled kernel settings λ=0.2, σ=2 and s=1. For “General” and “Viral collection” experiments, we also evaluated other combinations of the tuning parameters at the following values: λ={0.1,0.3}, σ={0.5,1,5,10,50,100}, and s={0.5}.

## 3 RESULTS

### 3.1 Edge case simulations

**Figure 1:**
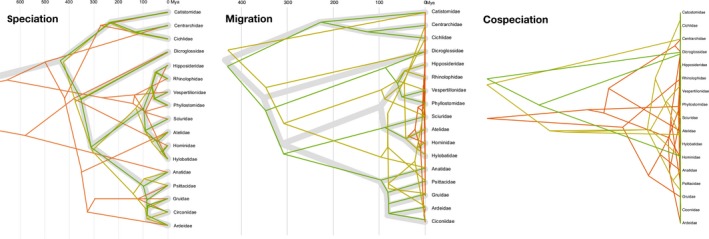
Effect of varying model parameters on simulated pathogen trees under edge case scenarios. The host phylogeny is displayed with broad gray branches. (left) Decreasing speciation rates Λ={1,0.04,0.001} for green, yellow, and red, respectively; *P* = 0, *M* = 0) results in a greater frequency of deep speciation events. (center) Increasing migration rates M={0.00055,0.004375,1} for green, yellow, and red, respectively; Λ=1, P=1) results in a greater frequency of host switching events. (right) Decreasing cospeciation rates P={1,0.50,0.25} for green, yellow, and red, respectively; $\Lambda =10^{-6}$, *M* = 0) results in a greater frequency of deep speciation events. Here, the host tree has been purposely left out of the plot because of much shorter timescale compared to the pathogen trees

We implemented a reverse‐time model to simulate pathogen trees, given a fixed host tree and coevolutionary parameters: the within‐host speciation (lineage duplication) rate, Λ; migration rate, *M*; and cospeciation probability, *P*. To examine the response of different distance measures (Table 1) to variation in these coevolutionary parameters, we initially adjusted each parameter individually while holding the others constant (Table S1). The purpose of these edge case simulation experiments was to verify the expected effect of each model parameter under extreme conditions where their expected influence on pathogen tree shape was unambiguous. We also used these experiments to establish the potential for various distance measures to extract information about cospeciation processes by comparing the shapes of host and pathogen trees. To examine how pathogen tree shapes responded to changes in each model parameters, we plotted pathogen and host trees together for a small number of parameter values per edge case scenario (Figure 1).

Under the “speciation only” scenario, we varied the speciation rate Λ while fixing the migration rate *M* and cospeciation probability *P* to 0. Decreasing Λ led to a greater chance of duplication events where multiple pathogen lineages coexist in an ancestral host species (Figure 1, left panel). Conversely, high values of Λ resulted in high concordance between pathogen and host trees. In the “migration only” scenario, we varied *M* and fixed *P* to 1 and Λ to 1, the highest rate that we evaluated in this study. Increasing *M* resulted in greater discordance in shape between the host and pathogen trees as pathogen lineages switched into other hosts and immediately speciated with the extant pathogen species, which also compressed the timescale of the pathogen tree (Figure 1, central panel). Finally, we varied *P* in the “cospeciation only” scenario with *M* set to 0 and Λ set to 10^−6^. Setting Λ to the lowest value exaggerated the effect of reducing *P*, since any pathogen lineages that did not cospeciate with the host became free to speciate on a much longer timescale (Figure 1, right panel).

**Figure 2:**
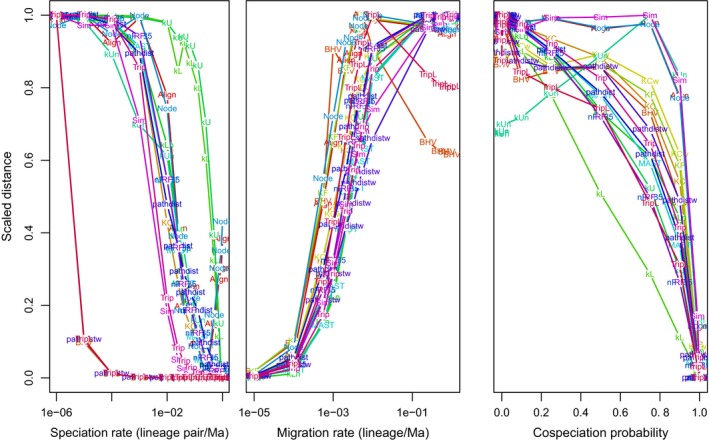
Summary of associations between tree distance measures and model parameters. Each point represents the mean distance measure (text label) for 100 replicate simulations, rescaled to range from 0 to 1 to facilitate comparisons between different measures. In the left panel, BHV, KF, pathdistw, KCw, and TripL almost perfectly overlap making it difficult to be distinguished

Next, we evaluated the response of the various distance measures to individually varying the parameters within each of these three scenarios, taking into consideration the means of the distance measures for the 100 simulated pathogen trees per combination of parameters (Figure 2). We observed substantial variation among the different tree distance measures (each scaled to their respective empirical range) in response to the within‐host speciation rate, Λ (Figure 2, left panel), migration rate (Figure 2, central panel), and cospeciation rate (Figure 2, right panel). We characterized this variation by the approximate Λ, *M*, and *P* values where the trends crossed a scaled distance of 0.5 ($\Lambda_{50}$, $M_{50}$, $P_{50}$, respectively). For the majority of distance measures (RF, nPH85, MAST, Align, Node, kLn, kUn, pathdist, KC), the $\Lambda_{50}$ was about 0.02/pair/Ma (hereafter Ma=million years ago). The unnormalized kernel measures kU and kL were more responsive at higher speciation rates ($\Lambda_{50}\approx 0.4$). Trip and Sim were responsive to lower rates ($\Lambda_{50}\approx 0.003$) and TripL, BHV, KF, KCw, and pathdistw changed only when Λ was very low ($\Lambda_{50}\approx 3.5\times 10^{-6}$). Most of the distances displayed an approximately monotonic relationship with Λ except for Node and Align, which both increased in distance as Λ approached 1. Node, Align, KF, and BHV were more responsive to slightly lower rates of migration ($M_{50}=10^{-4}$) than the other distances. All the distance measures displayed a monotonic relationship with *M*, with the exception of TripL, BHV, Align, Node and KF, which switched around *M* = 0.01/lineage/Ma. Finally, all measures were more responsive to higher values of *P* (0.8–1.0) but with more variation in their response to this parameter than *M*. For example, kUn and BHV sharply declined as *P* approached 1, whereas the other measures displayed a more gradual decline with *P*. kL was the only measure that displayed a roughly linear decrease of scaled distance with *P*. In case, the mean response of measures to model parameters masked excessive variation among replicate simulations, which would compromise the informativeness of the measure, we quantified the coefficient of variance (CV, ratio of standard deviation to the mean) from the same data (Figure S4). First, we observed that CVs were near zero for migration rates above 10^‐3^ and increased exponentially below this value for all measures. This was likely caused by the low values obtained by all distance measures in the absence of migration destabilizing the CV ratio. We tended to obtain low CV values for cospeciation probabilities below 0.8, although the distance measures TripL, BHV, KF, KCw, and pathdistw (which incorporate branch lengths) resulted in substantially higher CVs in this range. Again, a sudden increase in CVs associated with cospeciation probabilities near 1 was associated with low distances across measures. We obtained similar results for the edge cases varying speciation rates, except that the CVs tended to destabilize at rates above 0.01/lineage pair/Ma.

### 3.2 Simulation – hypercube sampling

**Figure 3:**
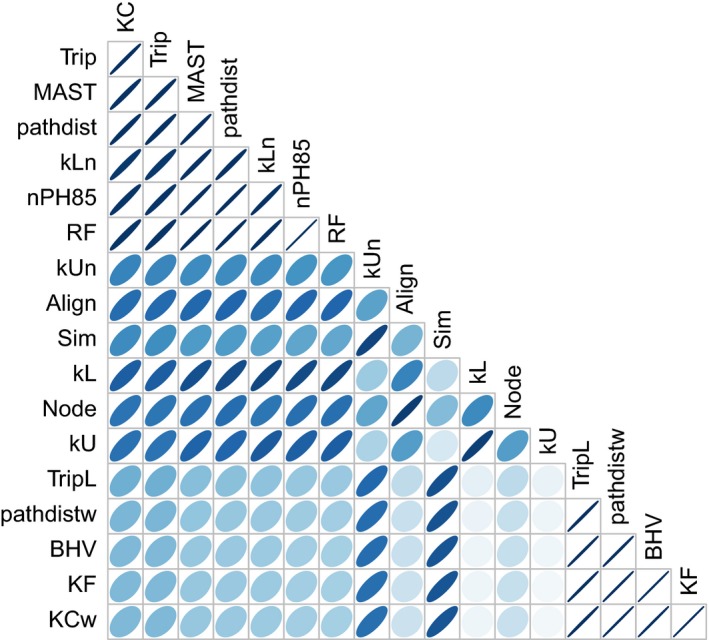
Summary of correlation matrix of tree distance measures. Spearman's rank correlations were calculated for 10,000 trees simulated under varying model parameter settings, narrower and blue‐darker is the bubble, higher is the correlation

For 500 different points sampled evenly from the parameter space defined by Λ, *M* and *P*, we used the nested speciation model to simulate 100 pathogen trees on the phylogeny relating hosts of viruses in the HBV family, for a total of 50,000 simulations. We emphasize that unlike the previous set of experiments, these simulations jointly varied all three model parameters. Next, we computed the distance measures in Table 1 for every simulated tree to the “observed” host tree, and averaged the distances for each of the 500 parameter settings. Figure 3 summarizes the nonparametric (Spearman's rank‐order) correlation tests for all pairs of distance measures. We observed strong correlations (*ρ* > 0.95) among a group of the distances comprising KC, Trip, MAST, pathdist, kLn, nPH85, and RF, and a second group of distances comprising TripL, pathdistw, BHV, KF, and KCw. The first group, characterized by an emphasis on tree topologies, was strongly correlated with kL as well (0.82< *ρ* < 0.90). Conversely, the second group was characterized by an emphasis on branch lengths. We also observed very high correlations between Align and Node (*ρ* = 0.94) and the two unnormalized kernel measures kU and kL (*ρ* = 0.94). Interestingly, Trip and Sim, which had the same response in edge case experiments for Λ and *M*, did not have a strong positive correlation (*ρ* = 0.62).

**Figure 4:**
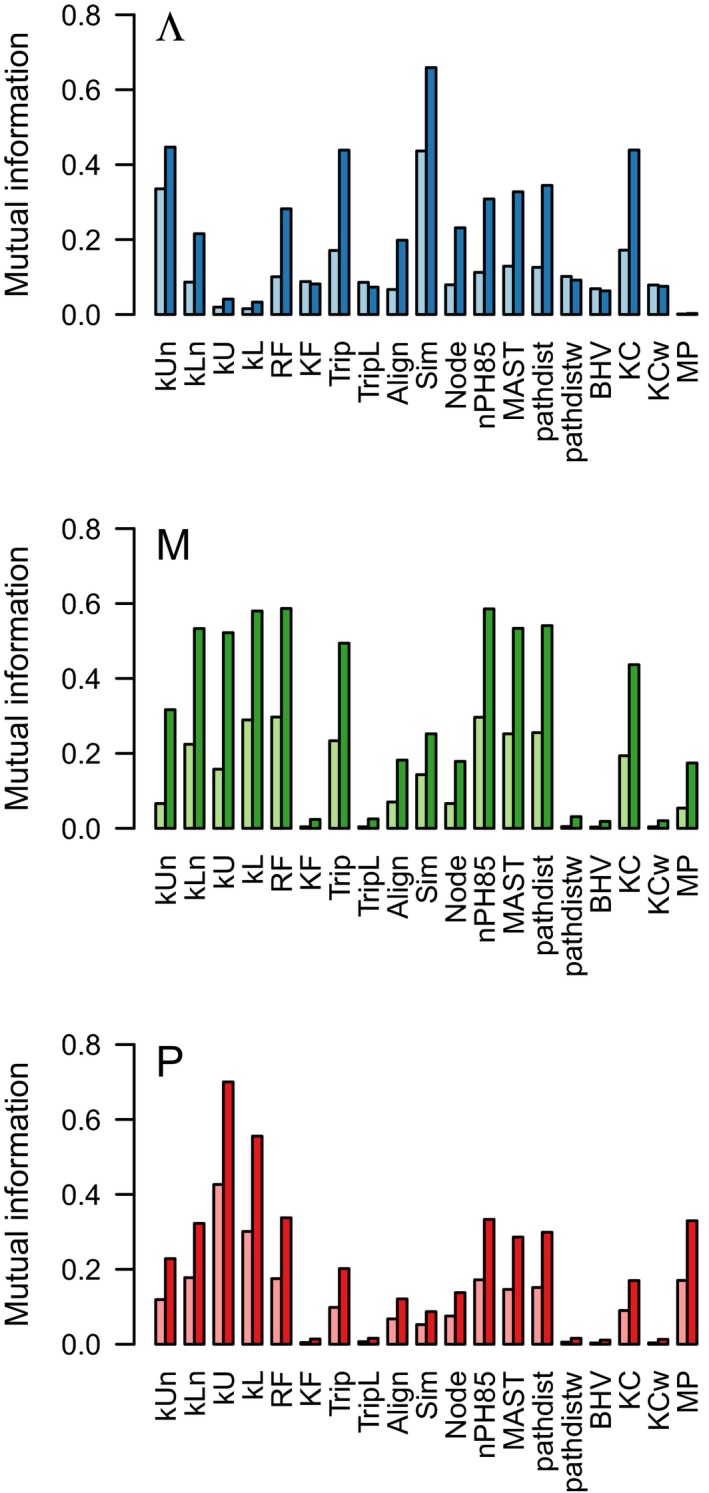
Barplots summarizing the mutual information of distance measures on the cophylogeny model parameters. The mutual information *I* was calculated by discretizing each distance *d* and model parameter θ into 10 bins respectively, for a total of 100 bins in the joint distribution p(d,θ), and then computing the sum $\sum_{i}\sum_{j}p\left(d_{i},\theta_{j}\right)\log\left(p\left(d_{i},\theta_{j}\right)/\left(p\left(d_{i}\right)p\left(\theta_{j}\right)\right)\right)$. If I=0, then d is independent of θ. Two values of *I* were computed for each distance. The left values were computed from the entire parameter space, whereas the right values were constrained as follows: (Λ) low speciation, *M* < 10^−4^; (*M*) high cospeciation *P* > 0.8, and; (*P*) low migration, *M* < 10^−4^. MP, Maximum Parsimony reconciliation

We used the mutual information to quantify the information content of each measure with respect to the three model parameters. Mutual information (MI) quantifies the information that we gain about a variable given that can only observe a second variable that may be associated with the first. For instance, it is commonly used to detect coevolution in genetic sequences (e.g., Dunn, Wahl, & Gloor, 2007). Based on our preliminary results with the edge case scenarios, we also calculated a second set of MI values where the parameter space was constrained to *P* > 0.8 for *M* and Λ; and by *M* < 10^‐4^/lineage/Ma for Λ and *P* (Figure 4). Overall, Sim was the most informative measure for Λ, while kU and kL were the most informative for *P*. Several measures obtained similar levels of MI for *M*, including kL, RF, and nPH85. Here, we included MP reconciliation analysis as well to compare it with the other distance measures; MP number of cospeciation events where evaluated with our cospeciation event *P*, MP number of transfer events was evaluated against *M* and MP number of duplication events against Λ. MP reconciliation obtained a relatively low level of informativeness for all the parameters.

**Figure 5:**
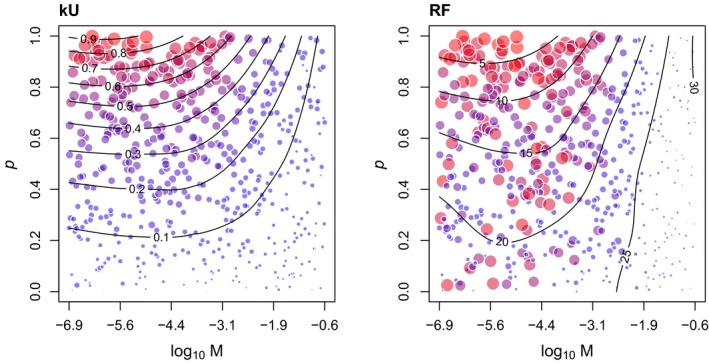
Contour plots summarizing the response of the unlabeled kernel (kU) and Robinson‐Foulds distance (RF) to variation in cospeciation probability (*P*) and migration rate (*M*). Each point represents the average of 100 replicate simulations for a given parameterization of the cophylogeny model. The area and coloring of points is proportional to the distance measure

**Figure 6:**
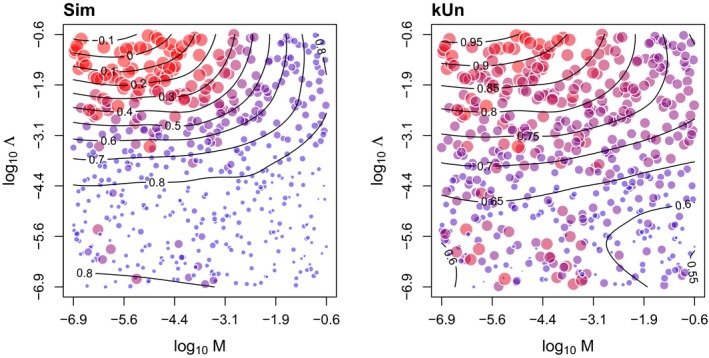
Contour plots summarizing the response of the Sim distance and normalized unlabeled kernel (kUn) to variation in speciation (Λ) and migration (*M*) rates. Each point represents the average of 100 replicate simulations for a given parameterization of the cophylogeny model. The area and coloring of points is proportional to the distance measure

To examine the response of kU to variation in *P* and *M* more closely, we generated contour plots for this measure and the popular RF distance for comparison (Figure 5). These plots clearly illustrate that the information content of either measure on *P* is dependent on the migration rate, and decays as *M* becomes too high. We note that unlike Figure 1, where the speciation rate was fixed, these contour plots mask extensive variation in Λ among simulations. Similarly, Figure 6 illustrates the response of the measures Sim and kUn to variation in Λ and *M*. Again, the information content of either measure on Λ decays when *M* becomes too high; this effect is more conspicuous for Sim.

### 3.3 Application to real data sets

Our simulation experiments reveal that the different distance measures respond differently to variation in speciation, migration and cospeciation rates. Furthermore, none of the distance measures is independently capable of conveying substantial information about all three cospeciation parameters. Although the simulated data provide a “ground truth” to these parameters, the underlying model relies on unrealistic assumptions (see Discussion section) that limit the biological realism of these data. To assess the response of these distance measures to phylogenies reconstructed from actual data, we collected published trees or sequences for matched sets of host and pathogen species from the literature. We searched the literature for studies of host‐pathogen cospeciation where the system was qualitatively described as having high or low levels of phylogenetic concordance due to cospeciation (the “General” collection, Table 2). We used these descriptions to partition the “General” collection into two categories.

**Figure 7:**
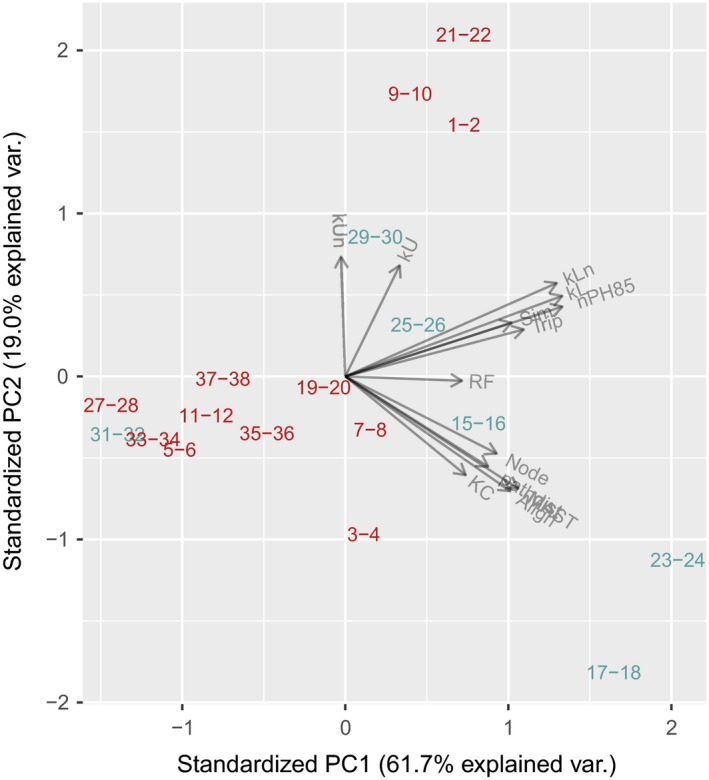
Biplot of a principal components analysis on distance measures for the general dataset. Each label represents a pair of host and pathogen trees that were characterized in the respective sources as cases of high (red) or low (blue) codivergence (see also Table 2). The gray vectors represent the variable loadings for the respective distance measures

This transition from simulated to actual data highlighted significant obstacles in the use of distance measures to cophylogeny studies. First, the measures often require the trees to be the same size, i.e., to have equal numbers of tips (Table 1). Distance measures that utilize labels, such as the RF distance, also require that the trees have the same labels, e.g., that the trees are alternative models for relating the same taxa. When simulating the data sets, it was trivial to generate pathogen trees that matched the labels of the host tree by initializing a single pathogen lineage in each host species. The biological reality of host–pathogen associations is frequently more complex, however. A pathogen species may be found in more than one host species, and a host species may be associated with multiple pathogen species. These cases may be accommodated by grafting additional branches with zero lengths to tips with multiple associations, to enforce a one‐to‐one map between the host and pathogen phylogenies (Geoghegan et al., 2017). Similarly, we grafted zero‐length branches to equalize the numbers and labels of tips in each pair of trees in the “General” data collection, and then calculated tree distance measures for each pair. When examining each distance measure individually, we did not observe any clear separation between high‐ and low‐codivergence tree sets in the “General” collection (Figure S1). This is consistent with findings from our simulation analysis that the distance measures vary substantially in their response to different cospeciation parameters. We next used a principal components analysis to examine the joint distribution of the general collection as a biplot. Because the number of dimensions (distance measures) equaled the number of observations (Lee, Zou, & Wright, 2010), we excluded distances with consistently low mutual information (<0.1) in our simulation experiments over the focused parameter space (*P* > 0.8, *M* < 10^−4^), viz., KF, TripL, BHV, KCw and pathdistw. The resulting projection (Figure 7) appeared to separate cases of low and high codivergence, respectively, with the exception of a cluster of high‐divergence cases (1–2, 9–10 and 21–22) and one low‐divergence case outlier (31–32). We note that the trees in 31–32, located in the midst of high‐divergence cases, had the fewest tips of any case (*n* = 5 for both hosts and parasites, Table 2), which suggests this outcome was affected by sampling variation. The alignment of loadings among the different distance measures in the biplot was consistent with our correlation analyses (Figure 3). Similarly, the high variable loadings on the first component of the biplot – combined with our simulation results – suggest that the characterization of phylogenetic concordance in these studies is strongly influenced by host switching (migration) events.

One of the unique features of the kernel methods compared to the other distance measures in this study is that they can be applied to unlabeled trees. This enables us to not only compute a distance between a pair of host and pathogen trees, but we can also compute distances between a host tree and pathogen trees from other pairings. In other words, it is not possible to compute the RF distance between the trees relating crinoids (sea lilies, Lanterbecq, Rouse, and Eeckhaut (2010), from couple 21–22) and the gut bacteria of termites (Noda et al. (2007), from couple 37–38) even though these trees are the same size. We can therefore embed all the trees into a common feature space defined by a given kernel (Figure S2). We exploited this characteristic to test whether pairs of trees in the general collection were significantly closer together in this feature space than expected by chance with a randomization test. We drew 18 random pairings of host and pathogen trees from the “General” collection, calculated the mean unlabeled kernel score (kU), and repeated this procedure to obtain 1,000 replicate means to approximate a null distribution. The mean kernel score for the actual tree pairs (*E*(*kU*) = 0.90) was located in the 99.9 percentile of this distribution, indicating that the actual pairs were significantly closer in the kU‐defined space than expected by chance ($p=2.0\times 10^{-4}$).

Finally, we examined a second collection, the “Viral,” of host and pathogen trees corresponding to 19 different virus families from a previous study (Geoghegan et al., 2017). Since the host trees were derived from a common time tree, we generalized the host tip labels to the family taxonomic level, making it feasible to compare nonassociated trees with both unlabeled and labeled kernels. In addition, branch lengths in the host trees were scaled in time to millions of years, whereas the pathogen trees were scaled to evolutionary time (expected numbers of nucleotide substitutions). This difference made it necessary to renormalize branch lengths in both host and pathogen trees (dividing by mean branch length) for kernel‐mediated comparison (kUn and kLn). Figure S3 comprises two PCA plots from the analysis of the similarity matrices using the unlabeled (kUn, left panel) and labeled (kLn, right panel) kernel functions, respectively. Again, we ran randomization tests for this collection using either kUn and kLn. When we ignored labels in comparing tree shapes, the mean kernel score for the actual tree pairs was *E*(kUn)  = 0.78 and located in the 33.1 percentile (p=0.67) of a randomized null distribution, indicating that the actual pairs were not significantly closer in the kUn‐defined space than expected by chance. We obtained substantially different results with a labeled kernel: the mean score (E(kLn)=0.19) was located at the 99.9 percentile of the randomized distribution ($p=7.0\times 10^{-4}$), indicating that the actual pairs were significantly closer in this feature space than expected by chance. Geoghegan et al. (2017) previously reported that the phylogenies of DNA viruses and their hosts tended to be more concordant than RNA viruses, which was attributed to their relatively higher rates of cospeciation and lower rates of migration. Here we observed the same trend for families of DNA viruses, especially Hepadnaviridae, Poxviridae and Papillomaviridae. However, we also observed significant clustering for the RNA virus families Orthomyxoviridae and Potyviridae. In the latter case, clustering was most likely driven by the unique distribution of these viruses in plant host species. Using nonparametric Wilcoxon tests, we found no significant difference in kUn distances separating DNA or RNA virus trees from their respective host trees (*p* = 0.17), but significantly greater labeled (kLn) distances for RNA viruses (*p* = 0.02).

## 4 DISCUSSION

There is a deep literature on developing distance measures for the comparison of phylogenetic trees in order to quantify biological processes such as speciation (Kuhner & Yamato, 2014; Mooers, 1997). Multiple quantitative frameworks for the comparison of phylogenetic trees have also been developed for the study of cophylogeny, to determine whether the two sets of organisms share a coevolutionary history (e.g., Doyon et al., 2011; Huelsenbeck et al., 2000). We therefore anticipated extensive applications of tree distances in the literature for analyzing cophylogeny or coevolution. However, our survey on papers citing the tree distance measures found only eight studies that have made use of measures for coevolutionary studies (0.002% of all studies reviewed, Table 1), which is a surprising outcome given the similar objectives of the respective fields. Instead, the comparison of trees in coevolutionary studies had frequently relied on other methods where a tanglegram is either assessed qualitatively by the investigator, or analyzed with a reconciliation method, which is computationally complex for probabilistic reconciliation or requires a subjective assignment of cost functions to the respective coevolutionary events (e.g., cospeciation, host switching, and extinction) for parsimony‐based reconciliations. The general objective of our study was to assess the potential utility of distance measures for cophylogenetic studies by comparing different measures on simulated and real data sets.

In this study, we did not attempt to evaluate a comprehensive set of all available distance measures; however, we endeavored to evaluate measures in relatively common use, augmented with a small number of kernel‐based measures recently proposed by our group. In addition to the measures in our study, there is a large number of distance measures that can be constructed from summary statistics such as Sackin's index (Blum & François, 2005). A summary statistic reduces a tree down to a single number that quantifies a biologically significant aspect of tree shape such as asymmetry (Mooers, 1997). Thus, we can obtain a distance from a summary statistic by taking its difference between the host and pathogen trees. However, these summary statistics usually do not incorporate tip labels, placing greater emphasis on similarity in tree shapes, and they can be difficult to normalize for comparing pairs of trees with different sizes (Pompei, Loreto, & Tria, 2012). In addition, there are several spectral methods that can be applied to trees by interpreting these objects as graphs (Hendy & Penny, 1993; Lewitus & Morlon, 2015) and the further development of tree distances continues to be an active area of research (Colijn & Plazzotta, 2017; Kendall & Colijn, 2016). It is therefore not feasible to evaluate all possible distances and for our purposes, we have only evaluated a representative subset of distance measures, including commonly used measures such as the RF distance.

Simulation experiments are an essential step to evaluate the response of a measure to variation in the data because the underlying parameters are known without ambiguity. However, the inherent assumptions of the simulation model may limit our ability to extrapolate from that analysis to real applications. In this study, we have taken the unusual approach of simulating the pathogen trees backwards in time along a fixed host tree. Our motivation for this approach is that it is more efficient to start from “sampled” lineages and converge back in time to their common ancestors, then to simulate forward from a single ancestor and discard cases that are not compatible with the expected endpoints. Simulating the pathogen tree forward in time requires the model to parameterize lineage extinction events, and requires the user to discard a potentially large number of simulations that do not match the observed number of lineages in the present or simply go extinct before any lineages can become sampled. We decided to use a reverse‐time simulation approach to avoid the computational cost of running simulations that would eventually be discarded. In addition, our speciation parameter (lineage duplication) is effectively the net rate of speciation minus extinction, in comparison to their forward‐time equivalents. However, this approach makes it difficult to incorporate unobserved extinction events, although inferring these events is already difficult due to the sensitivity of extinction rate estimates to model misspecification (Rabosky, 2010). In addition, we made a simplifying assumption that a single pathogen lineage was sampled per host. This assumption constrained host switching events in our model to be complete, such that the parasite lineage in the new host species becomes a distinct species from the original lineage by the sampling time (Johnson, Adams, Page, & Clayton, 2003). Although it is straight‐forward to model the sampling of multiple pathogen lineages in a host species within our reverse‐time framework, we sought to minimize the complexity of the parameter space to evaluate in our simulation experiments. Similarly, we assumed complete sampling of all extant pathogen lineages.

Our simulation experiments neglect the uncertainty in reconstructing phylogenetic trees from observed data. In other words, we have applied the distance measures directly to the “true” phylogenies generated under varying model parameters. This was a necessary simplifying assumption to reduce the number of simulation parameters, including the length of the sequence alignment, extent of missing data, rate of evolution, and models of nucleotide substitution, insertions, and deletions. Our primary objective was to evaluate the relative utility of different distance measures under idealized conditions. One should expect that this additional uncertainty should generally reduce the information that any given distance measure contains about the underlying model parameters. The problem of reconstructing accurate phylogenies affects all reconciliation methods, although Bayesian methods are expected to be more robust by sampling trees from the posterior distribution. Thus, a possible and simple method to ameliorate phylogenetic uncertainty would be to apply distance measures to random samples of host and parasite trees from a Bayesian analysis, although this approach would face the same problem of slow convergence for large data sets.

Another significant drawback to using distance measures is that they are too simple, reducing the information content of phylogenetic trees down to numbers and thereby discarding potentially useful information. However, this drawback makes it even more important to determine which distance measures are more useful and how different measures can be most effectively combined to complement each other's strengths and weaknesses. Our simulation analyses of tree distance measures demonstrated that some measures were more informative than others with respect to specific coevolutionary parameters. For example, the Sim measure (Hein et al., 2004) was the most responsive to variation in speciation rates, and the unlabeled kernel (kU) to variation in cospeciation probabilities. In addition, we tended to observe lower levels of mutual information between the model parameters and the numbers of corresponding events reconstructed by maximum parsimony, in contrast to those obtained with distance measures. Of the three parameters, the highest mutual information obtained with maximum parsimony reconciliation was obtained for the cospeciation probability, which was comparable to the distance‐based methods on average.

The measures evaluated in this study were frequently correlated with each other, but the correlations were seldom so extreme that the measures were essentially redundant, e.g., the group comprising BHV, KF, pathdistw, KCw, and TripL, which compare both topologies and branch lengths (Figure 3). We also determined that distance measures were more informative about the model parameters when the underlying parameter values were not so extreme that the host tree has essentially no influence on the shape of the pathogen tree; i.e., when the migration (host switching) rate was too high, or when the cospeciation probability was substantially less than one and the pathogen speciation rate was near zero (Figure 1). These scenarios would make it difficult to meaningfully quantify cophylogeny by any method. If the host switching rate is exceedingly high, then the pathogen species are “cosmopolitan” and freely utilize whichever host species they encounter, which would negate any influence of cophylogenetic effects on the pathogen phylogeny. In the second scenario, the pathogen speciation rate is so low that pathogen lineages speciate on a much longer timescale than their hosts, making the distribution of speciation events independent of the host phylogeny. This scenario may arise when pathogen gene flow is unrestricted among host species (Johnson et al., 2003).

Next, we applied these measures to two collections of phylogenies that were reconstructed from actual biological data. In the “General” collection of coevolutionary studies across all taxonomic groups, we retrieved a total of 18 studies – including parasitic and symbiotic associations – where authors described the trees as having a high or low degree of concordance. Only six of these studies reported low concordance. These assignments were largely based on a subjective qualitative assessment of phylogenetic concordance, and there are no quantitative criteria that have been applied generally across taxa. Given the broad diversity of taxonomic groups being studied, it is unlikely that any one of the coevolutionary processes is consistently determining either outcome. It is also not feasible to determine with complete certainty how each process contributed to the varying levels of concordance across these empirical studies. Nevertheless, the projection of these trees into a parameter space defined by the distance measures revealed some clustering of studies reporting high concordance. This result suggests that investigators are describing concordance in a consistent way across different biological systems, and that these subjective assessments can be at least partly quantified using distance measures.

Reconciliation methods implicitly assume that the pathogen phylogeny is the outcome of a stochastic process that has unfolded along the host phylogeny, shaped by events such as cospeciation or migration that have occurred at different rates. The distance‐based approach that we have evaluated in this paper is analogous to fitting a nonparametric model to the shape of the pathogen phylogeny, conditional on the host phylogeny – none of these processes is explicitly modeled by any of the distances evaluated in this study. Although many methods employ maximum parsimony to infer these events, the problem of reconciliation lends itself to probabilistic inference through maximum likelihood (Huelsenbeck, Rannala, & Yang, 1997) and Bayesian (Huelsenbeck et al., 2000) frameworks, which have already been developed for restricted scenarios, e.g., no speciation within hosts (Paterson & Banks, 2001).

The ideal Bayesian approach would be to jointly sample the host and pathogen phylogenies and reconstructions of coevolutionary events given the sequence and associational data – however, the enormous model space this would entail would likely limit this approach to small data sets. There is growing interest across disciplines in using simulation‐based methods, e.g., approximate Bayesian computation (ABC), to estimate parameters instead of directly calculating model likelihoods (Tavaré, Balding, Griffiths, & Donnelly, 1997). The basic premise of ABC is that fitting can proceed by adjusting the parameters of the model until it yields simulations that resemble the observed data. Although ABC is intuitively appealing and relatively straight‐forward to implement, it is challenging to find similarity measures for comparing simulated and observed trees that are efficient to compute and sufficiently informative to estimate the parameters. Baudet et al. (2014) recently used an ABC approach to cophylogeny using forward‐time simulation of pathogen trees on a fixed host phylogeny, and employed a single distance measure based on the number of tip labels shared between the largest isomorphic subtrees. Their results indicated a general lack of parameter identifiability, such that a given pair of trees can be explained equally well by a broad range of event combinations. In another recent paper, Alcala, Jenkins, Christe, and Vuilleumier (2017) applied multiple network statistics (e.g., degree size) to simulated tanglegrams to estimate host switching and cospeciation rates using a rejection ABC method. We anticipate that the analysis of distance measures presented here will provide an important foundation for the further development of ABC‐based methods as a promising approach to the study of cophylogeny. However, ABC is but one potential application of distance measures in this context. Studies of cophylogeny that involve sets of host and pathogen species often make a qualitative statement about whether the corresponding trees are concordant or discordant. Using distance measures to quantify the extent of discordance can provide an objective and reproducible framework to measure discordance that is comparable across systems and studies.

## CONFLICT OF INTEREST

None declared.

## AUTHORS CONTRIBUTION

MA and AP conceptualized the research, performed the bioinformatic experiments and wrote the manuscript. GN, YH, and MR implemented some of tree distance measures we used in R library Kaphi. BJ realized the python script for the Kernel label distance measure.

## Data Availability

Python script for simulation, data and R scripts for figures are available at our public GitHub repository (http://github.com/PoonLab/cophylo) and released to the public domain under the GNU General Public License (version 3).
